# Retraction: Salidroside inhibits the proliferation and migration of gastric carcinoma cells and tumor growth *via* the activation of ERS-dependent autophagy and apoptosis

**DOI:** 10.1039/d4ra90046d

**Published:** 2024-04-25

**Authors:** Wei Yan, Kai Li, Amin Buhe, Tianxiong Li, Peirong Tian, Jun Hong

**Affiliations:** a Department of General Surgery, Beijing Shijitan Hospital, Capital Medical University China wei_yan1@aliyun.com; b Department of Surgery, Vanderbilt University Medical Center USA

## Abstract

Retraction of ‘Salidroside inhibits the proliferation and migration of gastric carcinoma cells and tumor growth *via* the activation of ERS-dependent autophagy and apoptosis' by Wei Yan *et al.*, *RSC Adv.*, 2019, **9**, 25655–25666, https://doi.org/10.1039/C9RA00044E.

The Royal Society of Chemistry hereby wholly retracts this *RSC Advances* article due to concerns with the reliability of the data in the published article.

In Fig. 2A, the flow-cytometry images share similarities with data found in other publications by different authors (F. Gao, F. G. Wang, R. R. Liu, F. Xue, J. Zhang, G. Q. Xu, J. H. Bi, Z. Meng and R. Huo, *Int. J. Oncol.*, 2017, **50**(5), 1821–1831; X. Zhang, F. Gao, L. Zhou, H. Wang, G. Shi and X. Tan, *Oncol. Res.*, 2017, **25**(9), 1529–1541).

The image representing ‘Salid (Ctrl)’ in Fig. 2C is duplicated in a previously retracted publication by different authors (S. Shi, X. Hu, J. Xu, H. Lui and L. Zou, *RSC Adv.*, 2018, **8**, 19196–19207, Retracted on 20 Sep 2022, *RSC Adv.*, 2022, **12**, 26639–26639).

The ‘PCNA/Ctrl-SGC-7901’ panel in Fig. 5D is duplicated in another publication by different authors (Y. Zan, Z. Dai, L. Liang, Y. Deng and L. Dong, *Drug Deliv.*, 2019, **26**(1), 1080–1091).

The ‘Liver/Ctrl’ panel in Fig. 6C is duplicated in another publication by different authors, C. Ge, J. Xhang and F. Feng, *RSC Adv.*, 2019, **9**, 25022–25033.

The authors were asked to provide the raw data for this article, but did not respond. Given the significance of the concerns about the validity of the data, and the lack of raw data, the findings in this paper are not reliable.

The authors have been informed but have not responded to any correspondence regarding the retraction.

Signed: Laura Fisher, Executive Editor, *RSC Advances*

Date: 17/04/2024

## Supplementary Material

